# Synthesis and Characterization of Linear Copolymers Based on Pharmaceutically Functionalized Monomeric Choline Ionic Liquid for Delivery of *p*-Aminosalicylate

**DOI:** 10.3390/pharmaceutics15030860

**Published:** 2023-03-07

**Authors:** Shadi Keihankhadiv, Dorota Neugebauer

**Affiliations:** Department of Physical Chemistry and Technology of Polymers, Faculty of Chemistry, Silesian University of Technology, 44-100 Gliwice, Poland

**Keywords:** choline, *p*-aminosalicylate, poly(ionic liquid)s, anion exchange, drug delivery system

## Abstract

Bioactive linear poly(ionic liquid)s (PIL) were designed as carriers in drug delivery systems (DDS). Their synthesis was based on a monomeric ionic liquid (MIL) with a relevant pharmaceutical anion to create therapeutically functionalized monomers, which further can be used in the controlled atom transfer radical polymerization (ATRP). The presence of chloride counterions in the quaternary ammonium groups of choline MIL, e.g., [2-(methacryloyloxy)ethyl]trimethyl-ammonium chloride (ChMACl), was stimulated to undergo the anion exchange with *p*-aminosalicylate sodium salt (NaPAS) as the source of the pharmaceutical anion with antibacterial activity. The resultant [2-(methacryloyloxy)ethyl]trimethylammonium *p*-aminosalicylate (ChMAPAS) was copolymerized to attain the well-defined linear choline-based copolymers with various contents of PAS anions (24–42%), which were regulated by the initial ratio of ChMAPAS to MMA and conversion degree. The length of polymeric chains was evaluated by the total monomer conversion (31–66%) yielding degree of polymerization (DP_n_ = 133–272). Depending on the polymer carrier composition, PAS anions were exchanged by 60–100% within 1 h, 80–100% within 4 h, and completely after 24 h by phosphate anions in PBS imitating a physiological fluid.

## 1. Introduction

During the past two decades, a numerous drug delivery systems (DDS) have been investigated to improve bioavailability and administration of pharmaceutics [1,2,3,4]. In conventional medicine, when a drug’s dosage is lowered by early decay or its concentration is excessively high and harmful to cells it does not utilize all its therapeutic potential [5,6,7]. The combination of various strategies, including the application of polymeric carriers [8,9,10] with star-shaped [11,12,13], and grafted [14,15,16] topologies, have been investigated to improve drug effectiveness while minimizing its side-effects. A properly designed polymer structure with programmed activity allows extending the drug release time or/and for precise targeting of the place in the body where medicine should be delivered [17,18]. Biodegradability is an extra advantage, which is especially useful to decompose the polymer carrier in the organism when the therapeutic process is finished [19]. Another option is based on the stimulus-sensitive polymers, which are able to control drug delivery and release by responding to changes in the environment (usually pH, temperature, etc.) [20,21,22,23].

Polymers with ionizable groups are interesting options for intracellular administration of drugs due to the efficient destabilization of endosomal membranes, which is required to transport into the cytoplasm [24]. The specific group of ionic polymers is based on ionic liquids (ILs) that melt at temperatures below 100 °C, demonstrate thermal and chemical stability, lower vapor pressure, and show strong ionic conductivity. Additionally, they undergo anionic exchange, which can be employed for modification of physicochemical properties, for example, to change their solubility. These fascinating chemical substances with applications in a wide range of fields, have become popular in the areas of synthesis and catalysis, extraction, electrochemistry, analytics, biotechnology, etc., due to their highly customizable nature and outstanding capabilities [25,26]. Apart from their physical and chemical properties, ILs with biological activity are also attractive in the ecological, biochemical, and medical aspects [27,28].

Choline, that is, 2-hydroxyethyl trimethylammonium chloride, is one of the well-known bioILs with evident biocompatibility and bioavailability, as well as antibacterial activity properties, which are highly appreciated in DDSs [29]. Furthermore, choline is produced by the human body, where it plays vitamin-like functions and is employed in a variety of vital processes as a part of phospholipids, e.g., lecithin, or precursor of acetylcholine. In turn, when the counterion is a salicylate anion, then this compound exhibits anti-inflammatory properties and can be used in the acute treatment of sore throats. It also has an analgesic effect, and most importantly, it is well tolerated by the body. Therefore the choline-based ILs can enhance the pharmacodynamic and pharmacokinetic characteristics of the carried drug [30]. The commercially available choline ester derivative [2-(methacryloyloxy)ethyl]trimethylammonium chloride, known as methacryloylcholine (ChMACl), has been reported as a monomeric choline-based IL. This monomer demonstrates potential in the synthesis of polymerized ionic liquids (PILs) [31], which are successful carriers for delivery of pharmaceutical anions. In other cases, the choline-based PILs acted as the universal matrices, where chloride anions were convenient to exchange with anionic drugs, such as sulfacetamide [32], *p*-aminosalicylate (PAS), fusidate, clavulanate, and piperaciline [33,34,35,36], to generate the pharmaceutically active polymeric systems. However, the choline-based PILs have been also prepared by polymerization of choline IL monomer with pharmaceutical anions, such as salicylate [31,37,38], fusidate, and cloxacillin.

**Figure 1:**
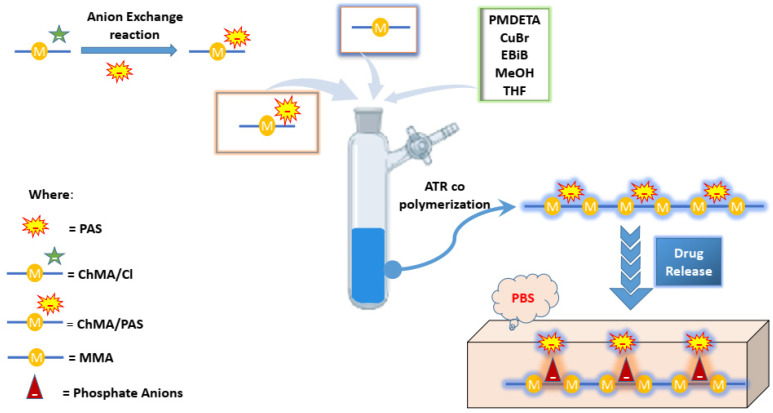
Schematic route of the ionic exchange of the monomer ChMACl to ChMAPAS and its use in the synthesis of the linear copolymer of ChMAPAS/MMA.

In the present study, the linear choline-based PILs with PAS anions (Figure 1) were synthesized using the pharmaceutically functionalized monomeric IL, that is [2-(methacryloyloxy)ethyl]trimethylammonium *p*-aminosalicylate (ChMAPAS). This new IL was obtained by the exchange of the chloride counterion in ChMACl with a PAS anion. The pharmaceutical anion was selected because PAS is an antibiotic which prevents *Mycobacterium tuberculosis* from multiplying and causes them to die. For this reason, it is mostly used to augment the activity of other anti-tuberculosis drugs and to avoid the drug-resistant effect [39,40,41]. The PAS content in the polymer was regulated by copolymerization of ChMAPAS and methyl methacrylate (MMA) in various proportions as well as using different ratios of monomer to initiator in the atom transfer radical polymerization (ATRP). Additionally, the P(ChMACl-*co*-MMA)s were synthesized to indicate the influence of pharmaceutical anion on the polymerization reaction and the structural parameters of the resulting polymers. The obtained PAS-containing polymers are ionic conjugates, in which the drug anion is connected by an ionic bond with a polymer matrix. Because of that, drug release is based on its exchange generated by phosphate anions in the PBS buffer, which was applied as a factor mimicking the natural environment of the physiological fluid during an in vitro study.

## 2. Materials and Methods

Methyl methacrylate (MMA, Alfa Aesar, Warsaw, Poland) was dried by molecular sieves under argon. [2-(Methacryloyloxy)ethyl]trimethyl-ammonium chloride (ChMACl, 80% aq. solution, Sigma-Aldrich, Poznan, Poland) was dried under reduced pressure until it reached a consistent weight. Copper (I) bromide (CuBr; Fluka, 98%, Steinheim, Germany) was purified by using glacial acetic acid and stirring, then filtered, washed in ethanol and diethyl ether, then dried under vacuum. Ethyl 2-bromoisobutyrate (EBiB), *N*,*N*,*N*′,*N*″,*N*″-pentamethyldiethylene-triamine (PMDETA), and phosphate-buffered saline (PBS) were purchased from Sigma-Aldrich (Poznan, Poland) and used as received. Methanol (MeOH, Chempur, Piekary Śląskie, Poland) and tetrahydrofuran (THF, Sigma-Aldrich, Poznan, Poland) were dried using molecular sieves under argon. Sodium *p*-amino-salicylate (NaPAS, 98%) was used without purification as received from Alfa Aesar (Warsaw, Poland).

### 2.1. Synthesis of [2-(Methacryloyloxy)ethyl]trimethylammonium ρ-aminosalicylate (ChMAPAS)

**Figure 2:**
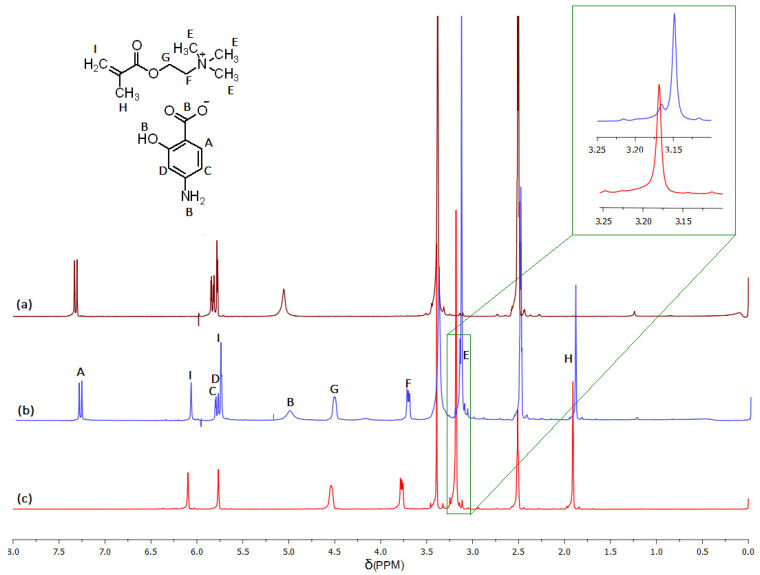
^1^H NMR spectra of the anion exchange of monomer, sodium salt of PAS (**a**), PAS after anion exchange (**b**), and ChMACl before anion exchange (**c**).

The vacuum-dried ChMACl (12 mmol, 2.5 g) was dissolved in 12.5 mL of MeOH (solution 1), then the salt of PAS (12 mmol, 2.54 g) was dissolved in 12.7 mL of MeOH and added to solution 1. The reaction mixture was stirred for 24 h in a dark place at room temperature until the precipitation of NaCl crystals (3 h) finished, and then the solution was filtered twice to remove the salt. The filtrate containing monomers with PAS anions was concentrated on a rotary evaporator at room temperature using a water pump and it was dried under reduced pressure until it became a powder product. Yield: 72%. ^1^H NMR (DMSO-d_6_,300 MHz, δ, ppm): 7.3 (1H, –CH in aromatic ring), 6.09 and 5.76 (2H, –CH_2_ in vinyl group), 5.6–5.8 (2H, –CH in aromatic ring), 5.02 (2H, –NH_2_), 4.52 (2H, –CH_2_–O–), 3.73 (2H, –CH_2_–N^+^–), 3.16 (9H, –N^+^(CH_3_)_3_), 1.90 (3H, –CH_3_) (Figure 2).

### 2.2. Synthesis of Linear Copolymer P(ChMAPAS-co-MMA) by ATRP (an Example for ChMAPAS/MMA (25/75) IA)

**Figure 3:**
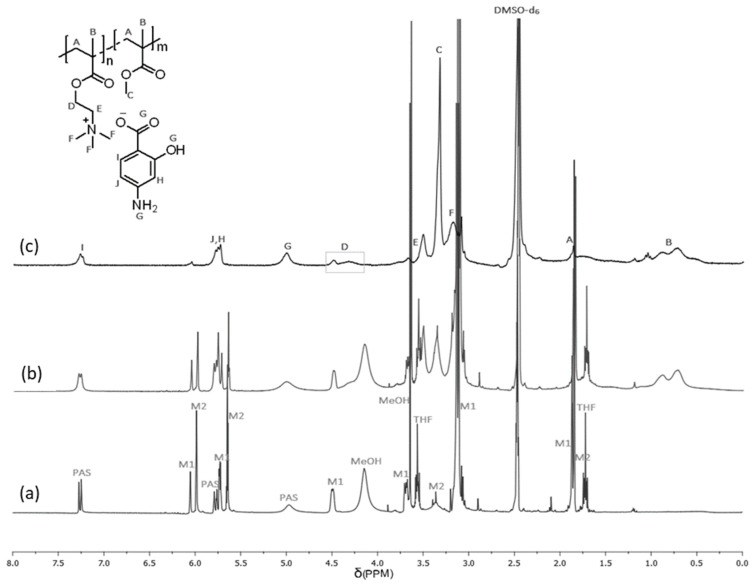
^1^H NMR spectra of reaction mixture in the synthesis of P(ChMAPAS-*co*-MMA) IA at start (**a**), and the end polymerization (**b**), and for the purified polymer (**c**).

In a Schlenk flask, ChMAPAS (1.5 mmol, 0.53 g) was dissolved in MeOH (0.58 mL), and then MMA (4.4 mmol, 0.47 mL), THF (0.18 mL), and PMDETA (0.015 mmol, 0.003 mL) were added. Next, the homogeneous mixture was degassed by two freeze–pump–thaw cycles, and then EBiB (0.015 mmol, 0.002 mL) as an initiator was introduced to the mixture, and the initial sample was taken out, and the freeze–pump–thaw cycle was repeated one more time. Afterward, CuBr catalyst (0.015 mmol, 0.002 g) was added to the mixture very quickly and the reaction flask was immersed in the oil bath at 40 °C. After 40 min, when a significant increase of the mixture’s viscosity was observed, the reaction was stopped. The sample was taken out to determine the monomer conversions, whereas the rest of the mixture was exposed to the air, subsequently it was dissolved in MeOH (1 mL) and twice precipitated in the chloroform–diethyl ether mixture to remove the catalyst from the polymer. The solvents were evaporated, and finally, the polymer was dried under reduced pressure. Yield: 62%. ^1^H NMR (DMSO-d_6_,300 MHz, δ, ppm): 7.3 (1H, –CH in the aromatic ring), 5.6–5.8 (2H, CH in the aromatic ring), 5.04 (2H, –NH_2_), 4.5–4.2 (2H, –CH_2_–O–), 3.6–3.4 (2H, –CH_2_–N^+^–), 3.45–3.25 (3H, –O–CH_3_), 3.3–2.9 (9H, –N^+^–(CH_3_)_3_), 1.8–1.5 (2H, –CH_2_), 1.25–0.3 (3H, –CH_3_) (Figure 3).

The syntheses of linear copolymers of ChMAPAS/MMA with the other molar ratios (IC: 50/50 and ID: 75/25) as well as with another ratio of monomer/initiator (IB: 600/1) were carried out according to the same procedure.

### 2.3. Synthesis of Linear Copolymer P(ChMACl-co-MMA) by ATRP (an Example for ChMACl/MMA (25/75) IIA)

**Figure 4:**
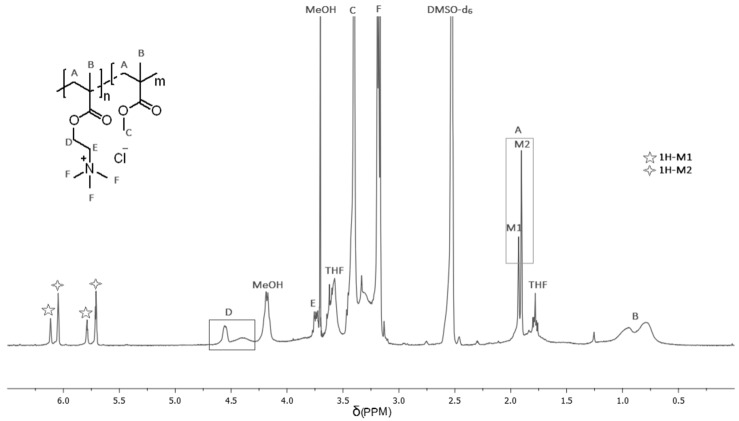
^1^H NMR spectra of P(ChMACl-co-MMA) IIB.

In a Schlenk flask, ChMACl (9.6 mmol, 2 g) was dissolved in MeOH (2 mL) and then MMA (28.9 mmol, 3.08 mL), THF (0.7 mL), and PMDETA (0.096 mmol, 0.02 mL) were added. After that, initiator EBiB (0.096 mmol, 0.01 mL) was introduced, and the mixture was degassed by three freeze–pump–thaw cycles. At the end, catalyst CuBr (0.096 mmol, 0.01 g) was added. Further procedure was performed as described above for the synthesis of P(ChMAPAS-*co*-MMA), but the reaction was stopped after 90 min. Yield: 68%. ^1^H NMR: (DMSO-d_6_, δ, ppm): 4.5–4.25 (2H, –CH_2_–O–), 3.7–3.5 (2H, –CH_2_–N^+^–), 3.5–3.25 (3H, O–CH_3_), 3.35–3.05 (9H, –N^+^–(CH_3_)_3_), 2.0–1.85 (3H, –CH_2_), 1.2–0.6 (3H, –CH_3_) (Figure 4).

The syntheses of linear copolymers of ChMACl/MMA with the other molar ratios (IIC: 50/50 and IID: 75/25) in addition to another ratio of monomer/initiator (IIB: 600/1) were performed regarding to the same procedure.

### 2.4. Drug Release Studies of Pharmaceutical Anion (PAS) by Polymer Carrier

The solution of linear copolymer was prepared from ChMAPAS (2 mg) dissolved in 2 mL of PBS with a pH of 7.4 (a water-based salt solution containing 10 mM sodium phosphate, 2.7 mM potassium chloride, and 137 mM sodium chloride, the osmolality and ion concentrations of the solutions match those of the human body). Then, a dialysis cellulose membrane bag (MWCO = 3.5 kDa) was filled with the 1 mL of polymer solution, and then placed into a glass vial containing 45 mL of PBS and stirred at 37 °C. The dialysis was carried out for 74 h. The progress of the drug release process was observed by a change in its concentration in the receival PBS solution outside the dialysis bag, from where samples with released drug (1 mL) were taken at several time points. The samples were analyzed by UV-Vis spectroscopy to determine the amount of release drug by measuring the absorbance maximum at λ = 265 nm for PAS^-^. The calculations of amount of the drug present in the release medium were performed on the basis of the Lambert–Beer law using the linear range of the calibration curve for the PAS solution in PBS. Each result is an average of three parallel measurements. After analysis, the sample was returned to the glass vial to maintain a constant volume of PBS medium. The kinetic profiles of the active substance release are presented as a dependency of the percentage amount of released drug by time.

### 2.5. Characterization

The ^1^H NMR spectra data were collected by a UNITY/NOVA spectrometer (Varian, Australia Pty, Mulgrave, Australia) operating at 300 MHz. The samples were prepared in deuterated dimethyl sulfoxide-d_6_ (DMSO-d_6_) as an appropriate solvent, and with the use of tetramethylsilane (TMS) as an internal standard for measurements. Size exclusion chromatography (SEC) was used to assess the dispersity index (Ð) and the average molecular weight (M_n_). The measurements were performed on an Ultimate 3000 chromatograph (Thermo Fisher Scientific, Waltham, MA, USA) with a differential refractometer RefractoMax 521 detector, at 40 °C in the water line. The polymer samples, prepared in deionized water, were passed through a precolumn TSKgel Guardian SuperMP(HZ)-H (4.6 mm × 2 cm, particle size of 6 μm) and two columns TSKgel SuperMilipore HZ-H (4.6 mm × 15 cm, particle size 6 μm) with a flow rate of 0.45 mL/min. The calculations were based on poly(ethylene oxide)/poly(ethylene glycol) (PEO/PEG) standards (982–227,000 g/mol). The drug content (DC) and the amount of released pharmaceutical anion of PAS in the in vitro studies were analyzed by ultraviolet–visible light spectroscopy (UV-Vis, spectrometer Evolution 300, Thermo Fisher Scientific, Waltham, MA, USA). The sample of polymer in PBS (0.05 mg/mL) was placed in a quartz cuvette and measured at λ = 265 nm. The calibration curve was obtained for a drug concentrations that ranged from 0.1mg to 0.006 mg/mL in PBS.

## 3. Results and Discussion

### 3.1. Synthesis of the Pharmaceutic Functionalized Monomer by Anion Exchange with PAS Salt

**Scheme 1:**
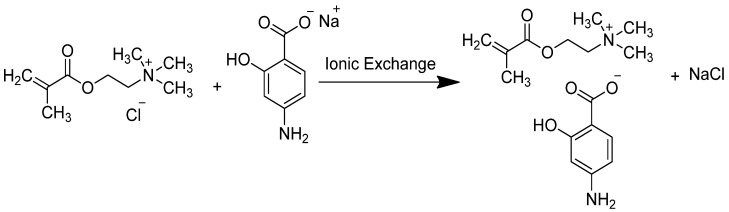
Ionic exchange of ChMACl by NaPAS to obtain ChMAPAS as the pharmaceutically biofunctionalized monomeric IL.

The water-soluble ionic liquid functionalized with polymerizable methacrylate group, i.e., [2-(methacryloyloxy)ethyl]trimethylammonium chloride (ChMACl), was modified with *p*-aminosalicylate sodium salt (NaPAS) via exchange of the Cl^-^ anion with PAS^-^ to produce a monomer containing a pharmaceutical anion, i.e., [2-(methacryloyloxy)ethyl]trimethylammonium *p*-aminosalicylate (ChMAPAS) as it is presented in the Scheme 1. The presence of PAS anions is advantageous for the future application in the treatment of respiratory system, including tuberculosis, bacterial sinusitis, acute otitis media, and chronic bronchitis, as well as for the primary treatment of inflammatory bowel disease [33]. The efficiency of exchange reaction in the monomer was calculated by the ^1^H NMR spectrum (Figure 2) indicating 77%. The shifting of signal E assigned to nine protons in trimethylammonium group from 3.18 ppm to 3.14 ppm because of the counterion change confirmed the incorporation of pharmaceutical anions in the monomer (see magnification in Figure 2).

### 3.2. Characterization of Linear Copolymers Obtained from ChMAPAS

**Scheme 2:**
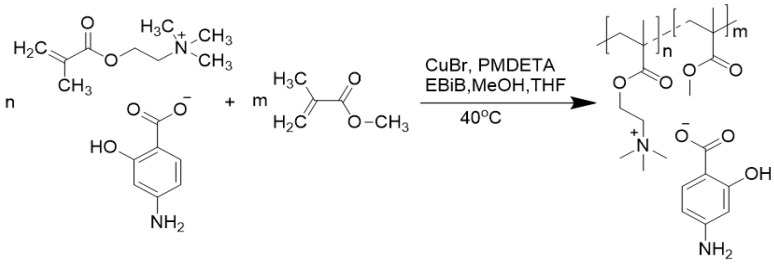
Synthesis of linear copolymer P(ChMAPAS-*co*-MMA) IA-D directly functionalized by pharmaceutical anions in via copolymerization of ChMAPAS and MMA.

The above-described monomer ChMAPAS was copolymerized with methyl methacrylate (MMA) in various proportions (25/75, 50/50 and 75/25) to produce linear copolymers P(ChMAPAS-*co*-MMA)s IA-D with various contents of the PAS drug. ATRP reactions were performed at 40 °C using EBiB as an monofunctional initiator, and CuBr/PMDETA catalytic system in MeOH/THF solvents (Scheme 2). The conversion of monomers into the polymer was monitored by ^1^H NMR (Figure 3). The calculation was based on the integration of a broad signal B (0.54–1.16 ppm) coming from protons of the methyl group in the main chain of the formed polymer and the signals of one proton in the vinyl group of unreacted ChMAPAS and MMA monomers (6.11–5.70 ppm). The conversion of ChMAPAS was determined using a proton of the vinyl group in the monomer (6.09 ppm) and a proton in the PAS anion present in both monomer and polymer (7.3 ppm). Additionally, the formation of the main chain of the polymer was confirmed by the appearance of the broad signal A at 1.9 ppm, corresponding to the methylene group. The series of analogical copolymers P(ChMACl-*co*-MMA)s IIA-D was synthesized at similar ATRP conditions as those for PAS-containing polymers to indicate the influence of counterions on the reaction rate. The representative spectrum of Cl-based copolymer is presented in Figure 4. Characterization of P(ChMAPAS-*co*-MMA)s IA-D and P(ChMACl-*co*-MMA)s IIA-D synthesized by ATRP are shown in Table 1.

**Figure 5:**
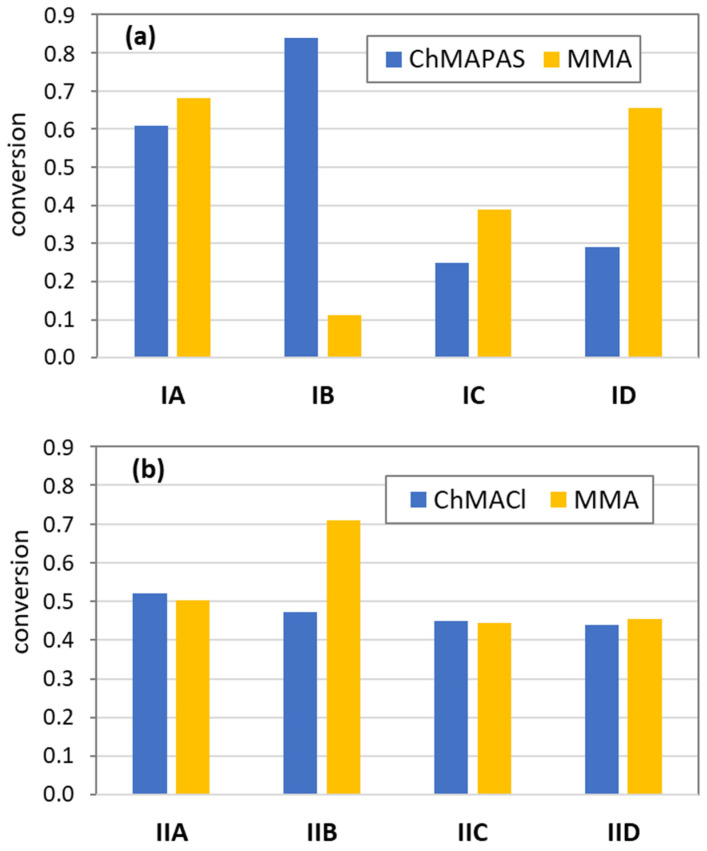
Comparison of monomer conversions in copolymerization of ChMAPAS/MMA (**a**) and ChMACl/MMA (**b**).

The value of DP_n_ corresponding to the length of polymeric chains shows that they were shorter in the case of ChMAPAS copolymers, which were obtained twice as quickly because of the higher viscosity of the reaction mixture than for analogous ChMACl copolymers, with exception for IA vs. IIA. In the case of PAS copolymers, the conversion of MMA was higher, and it was increased with the initial content of ionic monomer, whereas Cl-based copolymers were attained at similar conversions of comonomers independently on their initial ratios (Figure 5). The variety of relative reactivity ratio of ChMA vs. MMA between both series indicated the influence of the counterion type. The polymers prepared at the ratio of monomer to initiator 400/1 were characterized with the monomer conversion ranging 32–66% detected within a relatively short time of 40–125 min. At a higher initial ratio of monomer to initiator (600/1) used in synthesis of the copolymers IB and IIB, the lower rate of polymerization can be concluded due to significantly longer reactions yielding 31% and 65% of monomer conversion after 19 h and 5 h, respectively. Larger amounts of monomers in relation to the initiator in the reaction mixtures of IB and IIB had an influence on the specific behavior of the ionic monomer in relation to the MMA comonomer showing differences in conversions within the monomer pair for the studied system, i.e., X_ChMAPAS_ ≫ X_MMA_ (IB) vs. X_ChMACl_ < X_MMA_ (IIB). Comparing IB-IIB and IA-IIA with the same molar ratio of ChMA/MMA (25/75), but different monomer/initiator ratio (600/1 and 400/1, respectively), in the latter systems both monomers were incorporated into the polymer chains at similar conversions, i.e., X_ChMAPAS_ or X_ChMACl_~X_MMA_. At higher initial ratios of ChMA/MMA monomers, that is 50/50 (systems C), and 75/25 (systems D), differences in correlation of monomer conversions in the systems I with ChMAPAS were observed, showing a trend for increasing MMA conversion (X_ChMAPAS_ < X_MMA_) with increasing initial content of ionic monomer (Figure 5). In the case of the systems IIC and IID with ChMACl, the conversions of both monomers were similar (X_ChMACl_~X_MMA_).

Additionally, the polymer IIB was distinguished by the highest chain length, whereas in the polymer IB, the high content of ionic fraction was achieved, although it was not predominant in the initial mixture. According to the physicochemical characterization by the SEC method, the dispersity indices (Ð) of polymers corresponding to their molecular weight distribution were relatively low for both series. It confirms that the well-defined linear copolymers with homogeneous chains were formed as the consequence of low content of the side reactions occurring during the well-controlled ATRP as the pseudo-living process. The slightly higher dispersity of PAS-based polymers (Ð =1.25–1.36 at DP_n_ = 133–272) can be explained by bigger steric hindrance than that presented by smaller Cl anions in monomers incorporated into polymers IIA-IID (Ð = 1.12–1.26 at DP_n_ = 178–390).

**Figure 6:**
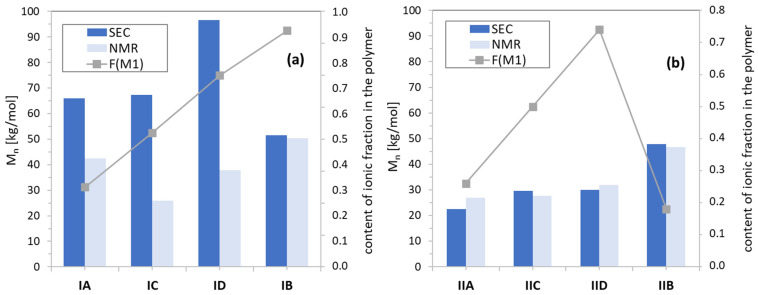
Dependence of M_n_NMR vs. M_n_SEC on content of ionic fraction in the series of PAS- (**a**) and Cl-based polymers (**b**).

Comparing the values of M_n_ by ^1^HNMR (calculated on the basis of monomer conversion) and M_n_ by SEC (based on the PEO calibration), different trends depending on the polymer series were observed. These values for the Cl-based polymers are similar, as shown in Figure 6b. The presence of pharmaceutical anions with steric hindrance changed the behavior of polymer chains in solution showing the discrepancy between M_n_NMR and M_n_SEC (M_n_NMR/M_n_SEC = 0.4 for IC-D and 0.6 for IA), which was reduced at the highest ionic content in the polymer IB (F_M1_ = 0.74) reaching M_n_NMR~M_n_SEC (Figure 6a). Additionally, both IA and IB obtained at 25% of initial content of ChMAPAS monomer were characterized by relatively long chains with DP_n_ ≥ 190, but they differed with the content of ChMAPAS units in the polymer (0.25 vs. 0.74, respectively). This effect can be explained by various composition of the formed chains generated by the initial proportions of monomer/initiator, which had an influence on the relative reactivity of comonomers yielding the subsequent tendency in the monomer conversions, that is X_ChMAPAS_~X_MMA_ in IA and X_ChMAPAS_ ≫ X_MMA_ in IB with the initial excess of nonionic monomer. All these factors are responsible for differences in the hydrodynamic volume of polymeric chains in solution. Furthermore, it should be mentioned that the PEO standards used in the calibration of SEC are hydrophilic but non-ionic polymers, which also impacted the determined M_n_SEC values.

**Figure 7:**
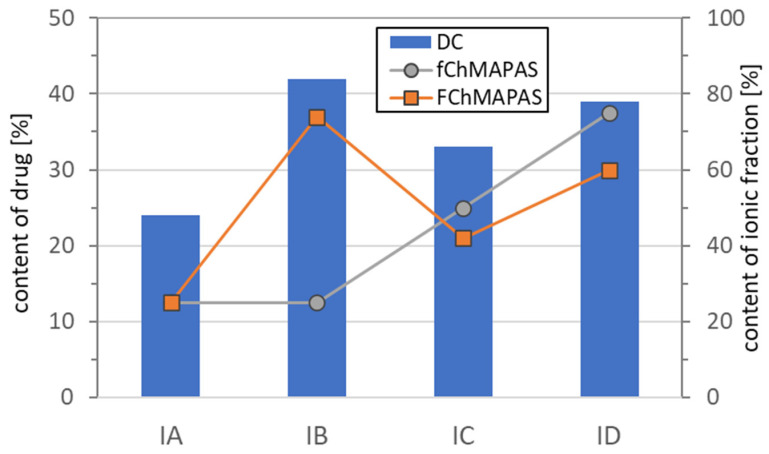
Comparison of ionic fraction content (f_ChMAPAS_, F_ChMAPAS_) by NMR and drug content (DC) by UV-Vis in linear copolymers.

The percentage amount of PAS included in the copolymer as the drug content (DC) was evaluated by UV-Vis spectroscopy. The amount of PAS, which was introduced by polymerization of the pharmaceutically functionalized monomer (ChMAPAS), was increased with the content of ionic units in the polymer (Figure 7). DC values ranged at 24–42%, but polymer IC seems to be the most advantageous system because the amount of drug contained in the polymer (DC = 33%) was well correlated with the initial content of PAS monomer in the reaction mixture and PAS unit content in the polymer (f_ChMAPAS_ = 50% and F_ChMAPAS_ = 42%). Additionally, the use of initial monomer ratio with a dominant amount of ChMAPAS (ID) did not guarantee the proportionally larger content of drug in the polymer (DC = 39%), which was limited by steric hindrance of the pharmaceutical anion in the monomer. Another effective option to attain similar drug content (DC = 42%) was the more diluted reaction mixture IB by the initial ratio of monomer to initiator (600/1) at the initial proportion of ChMAPAS/MMA equal to 25/75 which was beneficial for almost full conversion of the ionic monomer.

### 3.3. Drug Release

**Figure 8:**
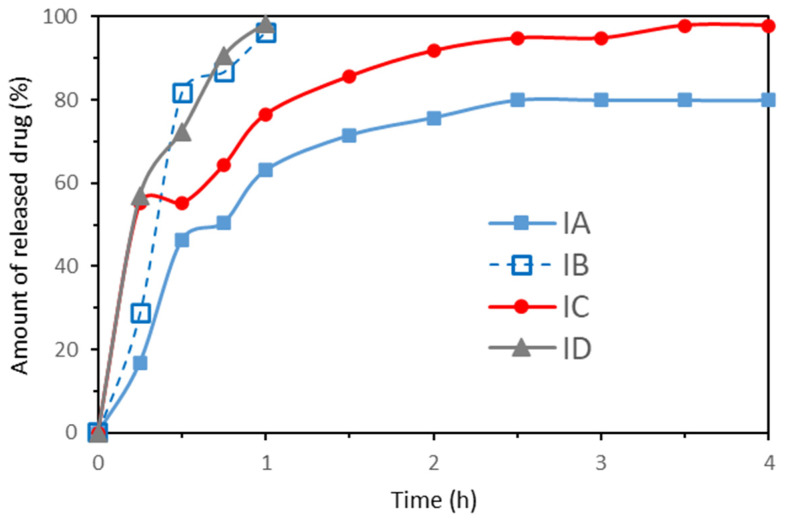
Drug release profiles for PAS-based copolymers in PBS at pH = 7.4 and 37 °C.

The *in vitro* drug release was measured using UV-Vis to monitor the exchange of PAS anions with the phosphate anions in the PBS solution. The kinetic profiles demonstrated a dramatically high rate of PAS, especially for samples ID and IB, where above 80% was released within one hour (Figure 8). The specificity of these systems was correlated with the compositions of polymers obtained at the highest initial ratio of ChMAPAS/MMA leading to a significantly higher conversion of MMA than ChMAPAS (ID) or with a higher ratio of monomer/initiator yielding an extremely high conversion of ionic monomer (IB). In those cases, higher rigidity of the formed chains appeared to be convenient for the increased repulsion effect, making the anions readily available during the exchange reaction by leaving the ionic group exposed to the environment. For the other systems IA and IC, the drug release was slower, removing 80–100% of PAS after 4 h, and the continuation of process for the sample IA until 24 h led to 100% of the final amount of released drug. These results confirmed that the release rate depends on several factors, including polymer structure, interactions between the bioactive anion and the polymer matrix, as well as between bioanions. However, the kinetic release profile for the IC polymeric carrier seems to be the most interesting for prolonged drug delivery due to the well-controlled release process and the effective amount of released PAS.

Previously, the P(ChMAPAS-*co*-MMA)s have been synthesized by anion exchange of P(ChMACl-*co*-MMA)s (containing 24–75% of ionic units), which have been obtained by polymerization of ChMACl and MMA by ATRP [34]. After polymer modification the introduced PAS anions were contained in 59–82%, whereas PAS was released in the amount of 33–45%. The same strategy (polymerization of chloride-based monomer and anion exchange in polymer) has been also applied for the graft copolymers, where the ionic units (13–46%) were distributed in the side chains, and the anion exchanged on the polymer resulted in a content of 31–64% PAS, and then its release at 18–42% after 48 h [34]. Comparing the values reported in the literature data with those described for the studied polymers, which were prepared by the reverse strategy, namely, anionic exchange in monomer and its polymerization, yielded polymers characterized with 24–42% of PAS content and complete PAS release, which is satisfactory for further studies on this type of polymeric carriers. In an analogous method, the polymers with salicylate anions have been synthesized, namely, by polymerization of salicylate-based monomers, which provided 43–85% of salicylate content and its release in ~50% not dependent on DC [37]. The slight difference between the structures of salicylate and PAS anions is related to the presence of an amino group in the *para* position of the aromatic ring in the latter drug, which has an influence on the reduction of PAS content in relation to salicylate, but it was beneficial in the release process to remove a significantly larger amount of PAS than salicylate.

## 4. Conclusions

The well-defined linear copolymers P(ChMAPAS-*co*-MMA)s with different ionic contents were designed to demonstrate their potential as carriers in DDS. The use of pharmaceutically active trimethylammonium-based IL monomer carrying a PAS counterion is convenient to synthesize the polymeric carriers with the adjusted amount of therapeutically active anion. The satisfactory content of PAS in the polymer matrix (24–42%) was corresponded to the efficient *in vitro* release of PAS (≥80%) in the PBS. However, the most optimal system was observed in the case, when the initial content of PAS monomer (50%) used in the polymerization mixture defined the content of PAS anions in the copolymer (33%) as well as the kinetic profiles indicated full drug release within 4 h. The strategy of PAS introduction in the monomeric IL by anion exchange and then its polymerization appeared to be advantageous to release larger drug amount at slightly lower drug content then it has been reported earlier for the analogous PAS systems, where the introduction of PAS by anionic exchange was carried out for the chloride-based polymers. The pharmaceutical activity of the selected PAS anion is crucial for the potential anti-bacteriostatic therapy, including the treatment of tuberculosis, but the investigated polymers can also be used to deliver other types of pharmaceutical anions, e.g., with anti-inflammatory effects.

## Figures and Tables

**Table 1:** Characteristics of P(ChMAPAS-*co*-MMA) IA-D and P(ChMACl-*co*-MMA) IIA-D synthesized by ATR (co)polymerization.

			^1^H NMR						SEC	
No	f_M1_/f_M2_ mol (%)	Time (h)	X_M1_ (%)	X (%)	DP_M1_	DP_n_	F_M1_ (%)	M_n_ (g/mol)	M_n_ (g/mol)	Ð
IA	25/75	0.66	0.61	0.66	68	272	0.25	42,500	65,900	1.29
IIA		1.5	0.52	0.51	52	203	0.26	26,900	22,400	1.26
IB	25/75	19	0.84	0.31	139	190	0.74	50,300	51,600	1.33
IIB		5	0.47	0.65	71	390	0.18	46,700	47,900	1.12
IC	50/50	0.91	0.25	0.32	56	133	0.42	25,800	67,300	1.25
IIC		1.7	0.45	0.45	90	179	0.50	27,600	29,600	1.13
ID	75/25	0.73	0.29	0.37	97	162	0.60	37,900	96,600	1.36
IID		2.1	0.44	0.44	132	178	0.74	31,900	29,900	1.14

IA–ID: M1 = CHMAPAS; IIA–IID: M1 = ChMACl; M2 = MMA; conditions: [M1+ M2]_0_:[EBiB]_0_:[CuBr]_0_:[PMDETA]_0_ = 400:1:1:1 (except IB and IIB where [M1 + M2]_0_:[EBiB]_0_ = 600:1), MeOH:ChMA = 1:1 (*v*/*w*), MeOH:THF = 3:1 (*v*/*v*), 40 °C; X_M1_—conversion of ChMAPAS or ChMACl, X—total conversion; DP_M1_—polymerization degree of ionic units; DP_n_—total polymerization degree; f_M1_, f_M2_—initial content of monomer in the reaction mixture; F_M1_—content of ionic fraction in the copolymer.

## Data Availability

Not applicable.

## References

[1] Coelho J.F., Ferreira P.C., Alves P., Cordeiro R., Fonseca A.C., Góis J.R., Gil M.H. (2010). Drug delivery systems: Advanced technologies potentially applicable in personalized treatments. EPMA J..

[2] Zhang Y., Chan H.F., Leong K.W. (2013). Advanced materials and processing for drug delivery: The past and the future. Adv. Drug Deliv. Rev..

[3] Gaurav I., Thakur A., Iyaswamy A., Wang X., Chen X., Yang Z. (2021). Factors affecting extracellular vesicles based drug delivery systems. Molecules.

[4] Jain K.K. (2008). Drug Delivery Systems.

[5] Visser J.G., Van Staden A.D.P., Smith C. (2019). Harnessing macrophages for controlled-release drug delivery: Lessons from microbes. Front. Pharmacol..

[6] Kingsley J.D., Dou H., Morehead J., Rabinow B., Gendelman H.E., Destache C.J. (2006). Nanotechnology: A focus on nanoparticles as a drug delivery system. J. Neuroimmune Pharmacol..

[7] Felice B., Prabhakaran M.P., Rodriguez A.P., Ramakrishna S. (2014). Drug delivery vehicles on a nano-engineering perspective. Mater. Sci. Eng. C.

[8] Pandey R., Khuller G. (2004). Polymer based drug delivery systems for mycobacterial infections. Curr. Drug Deliv..

[9] Kadajji V.G., Betageri G.V. (2011). Water soluble polymers for pharmaceutical applications. Polymers.

[10] Vega-Vásquez P., Mosier N.S., Irudayaraj J. (2020). Nanoscale drug delivery systems: From medicine to agriculture. Front. Bioeng. Biotechnol..

[11] Neugebauer D., Odrobińska J., Bielas R., Mielańczyk A. (2016). Design of systems based on 4-armed star-shaped polyacids for indomethacin delivery. New J. Chem..

[12] Mielanczyk A., Neugebauer D. (2015). Designing drug conjugates based on sugar decorated V-shape and star polymethacrylates: Influence of composition and architecture of polymeric carrier. Bioconjug. Chem..

[13] Bury K., Du Prez F., Neugebauer D. (2013). Self-assembling Linear and Star Shaped Poly (ϵ-caprolactone)/poly [(meth) acrylic acid] Block Copolymers as Carriers of Indomethacin and Quercetin. Macromol. Biosci..

[14] Maksym P., Neugebauer D. (2017). Self-assembling polyether-b-polymethacrylate graft copolymers loaded with indomethacin. Int. J. Polym. Mater. Polym. Biomater..

[15] Maksym P., Neugebauer D. (2016). Synthesis of amphiphilic semigrafted pseudo-Pluronics for self-assemblies carrying indomethacin. RSC Adv..

[16] Bury K., Neugebauer D. (2014). Novel self-assembly graft copolymers as carriers for anti-inflammatory drug delivery. Int. J. Pharmaceut..

[17] Golan D.E., Tashjian A.H., Armstrong E.J. (2008). Principles of Pharmacology: The Pathophysiologic Basis of Drug Therapy.

[18] Li D.C., Zhong X.K., Zeng Z.P., Jiang J.G., Li L., Zhao M.M., Yang X.Q., Chen J., Zhang B.S., Zhao Q. (2009). Application of targeted drug delivery system in Chinese medicine. J. Control. Release.

[19] Sung Y.K., Kim S.W. (2020). Recent advances in polymeric drug delivery systems. Biomater. Res..

[20] Chen K., Liao S., Guo S., Zheng X., Wang B., Duan Z., Zhang H., Gong Q., Luo K. (2020). Multistimuli-responsive PEGylated polymeric bioconjugate-based nano-aggregate for cancer therapy. Chem. Eng. J..

[21] Ofridam F., Ofridam F., Tarhini M., Lebaz N., Gagnière É., Mangin D., Elaissari A. (2021). pH-sensitive polymers: Classification and some fine potential applications. Polym. Adv. Technol..

[22] Ravi Kumar M.N., Kumar N. (2001). §, Polymeric controlled drug-delivery systems: Perspective issues and opportunities. Drug Dev. Ind. Pharm..

[23] Bhowmik D., Gopinath H., Kumar B.P., Duraivel S., Kumar K.S. (2012). Controlled release drug delivery systems. Pharma Innov..

[24] Yessine M.A., Lafleur M., Meier C., Petereit H.U., Leroux J.C. (2003). Characterization of the membrane-destabilizing properties of different pH-sensitive methacrylic acid copolymers. Biochim. Et Biophys. Acta (BBA)-Biomembr..

[25] Lu J., Yan F., Texter J. (2009). Advanced applications of ionic liquids in polymer science. Prog. Polym. Sci..

[26] Kowsari E. (2011). Ionic Liquids: Applications and Perspectives.

[27] Taha M., Almeida M.R., Silva F.A.E., Domingues P., Ventura S.P., Coutinho J.A., Freire M.G. (2015). Novel biocompatible and self-buffering ionic liquids for biopharmaceutical applications. Chem. A Eur. J..

[28] Fukaya Y., Iizuka Y., Sekikawa K., Ohno H. (2007). Bio ionic liquids: Room temperature ionic liquids composed wholly of biomaterials. Green Chem..

[29] Araújo J.M., Florindo C., Pereiro A.B., Vieira N.S., Matias A.A., Duarte C.M., Rebelo L.P.N., Marrucho I.M. (2014). Cholinium-based ionic liquids with pharmaceutically active anions. RSC Adv..

[30] Li X., Ma N., Zhang L., Ling G., Zhang P. (2021). Applications of choline-based ionic liquids in drug delivery. Int. J. Pharm..

[31] Bielas R., Bielas R., Mielańczyk A., Siewniak A., Neugebauer D. (2016). Trimethylammonium-based polymethacrylate ionic liquids with tunable hydrophilicity and charge distribution as carriers of salicylate anions. ACS Sustain. Chem. Eng..

[32] Bielas R., Siewniak A., Skonieczna M., Adamiec M., Mielańczyk Ł., Neugebauer D. (2019). Choline based polymethacrylate matrix with pharmaceutical cations as co-delivery system for antibacterial and anti-inflammatory combined therapy. J. Mol. Liq..

[33] Niesyto K., Neugebauer D. (2020). Synthesis and characterization of ionic graft copolymers: Introduction and in vitro release of antibacterial drug by anion exchange. Polymers.

[34] Niesyto K., Neugebauer D. (2020). Linear Copolymers Based on Choline Ionic Liquid Carrying Anti-Tuberculosis Drugs: Influence of Anion Type on Physicochemical Properties and Drug Release. Int. J. Mol. Sci..

[35] Niesyto K., Łyżniak W., Skonieczna M., Neugebauer D. (2021). Biological In Vitro Evaluation of PIL Graft Conjugates: Cytotoxicity Characteristics. Int. J. Mol. Sci..

[36] Niesyto K., Mazur A., Neugebauer D. (2022). Dual-Drug Delivery via the Self-Assembled Conjugates of Choline-Functionalized Graft Copolymers. Materials.

[37] Bielas R., Łukowiec D., Neugebauer D. (2017). Drug delivery via anion exchange of salicylate decorating poly (meth) acrylates based on a pharmaceutical ionic liquid. New J. Chem..

[38] Bielas R., Bielas R., Mielańczyk A., Skonieczna M., Mielańczyk Ł., Neugebauer D. (2019). supported poly (ionic liquid) graft copolymers as novel delivery systems of anionic pharmaceuticals for anti-inflammatory and anti-coagulant therapy. Sci. Rep..

[39] Donald P.R., Diacon A.H. (2015). Para-aminosalicylic acid: The return of an old friend. Lancet Infect. Dis..

[40] Minato Y., Thiede J.M., Kordus S.L., McKlveen E.J., Turman B.J., Baughn A.D. (2015). Mycobacterium tuberculosis folate metabolism and the mechanistic basis for para-aminosalicylic acid susceptibility and resistance. Antimicrob. Agents Chemother..

[41] Campregher C., Gasche C. (2011). Aminosalicylates. Best Pract. Res. Clin. Gastroenterol..

